# Conceptualizing researchers’ perspectives on involving professionals in research: a group concept mapping study

**DOI:** 10.1186/s12961-021-00685-2

**Published:** 2021-03-18

**Authors:** Christine E. Laustsen, Albert Westergren, Pia Petersson, Maria Haak

**Affiliations:** 1 Research Platform for Collaboration for Health, Faculty of Health Sciences, Kristianstad University, Kristianstad, Sweden; 2 Department of Health Sciences, Faculty of Medicine, Lund University, Lund, Sweden

**Keywords:** Ageing and health, Group concept mapping, Involvement in research, Professionals, Researchers

## Abstract

**Background:**

Researchers have shown an increased interest in involving professionals from outside academia in research projects. Professionals are often involved in research on ageing and health when the purpose is to address the gap between research and practice. However, there is a need to acquire more knowledge about what the involvement might lead to by exploring researchers’ experiences of involving professionals in research on ageing and health and developing conceptual areas. Therefore, the aim of this study was to identify conceptual areas of professionals’ involvement in research on ageing and health, from the perspective of researchers themselves.

**Methods:**

Group concept mapping, a participatory and mixed method, was used to conceptualize areas. Researchers with experience of involving professionals in research projects on ageing and health participated in qualitative data collection through brainstorming sessions (*n* = 26), and by sorting statements (*n* = 27). They then took part in quantitative data collection, where they rated statements according to how much a statement strengthened research (*n* = 26) and strengthened practice (*n* = 24). Data were analysed using multidimensional scaling analysis and hierarchical cluster analysis. In addition, a qualitative analysis of the latent meaning of the cluster map was conducted.

**Results:**

Analysis of the sorting stage generated five clusters illustrating conceptual areas of professionals’ involvement in research projects on ageing and health. The five clusters are as follows: complex collaboration throughout the research process; adaptation of research to different stakeholders, mutual learning through partnership; applicable and sustainable knowledge; legitimate research on ageing and health. The qualitative latent meaning of the cluster map showed two themes: the process of involvement and the outcome of involvement*.* A positive strong correlation (0.87) was found between the rating of strengthened research and practice.

**Conclusions:**

This study reveals conceptual areas on a comprehensive and illustrative map which contributes to the understanding of professionals’ involvement in research on ageing and health. A conceptual basis for further studies is offered, where the aim is to investigate the processes and outcomes entailed in involving professionals in research on ageing and health. The study also contributes to the development of instruments and theories for optimizing the involvement of professionals in research.

## Background

Researchers in the field of ageing and health have shown an increased interest in involving professionals from outside academia in different steps in the research process, with the aim of making research more relevant, applicable and sustainable [1–3]. Researchers also need to satisfy requirements from research funding bodies to involve people from outside academia, where the goal is to ensure quality, and to make good use of research by promoting collaboration in the research process [4–6]. Aspects researchers may be required to consider, describe and reflect on in grant proposals include societal relevance, collaboration and knowledge dissemination [4]. Although there is agreement between researchers, policy-makers and research funding bodies that people from outside academia should be involved in research, it is more difficult to agree on how and why they should be involved [7, 8], as this is an area which is still under development.

A variety of motives and underpinning philosophical assumptions affect why, how and when researchers involve and collaborate with different people in the research process [9]. Involvement can be seen as an umbrella term covering a continuum extending from information and education to collaboration [10]. However, a common feature is that the research is conducted *with* people and not *on* people [11].

Professionals constitute a specific group of people who may be involved in research studies. Professionals can be described as people with specific knowledge and sometimes even scientific knowledge gained through education [12, 13]. They may be interested in or stand to benefit from research in relation to their work, and their knowledge and expertise in relation to their work can contribute to research [14, 15]. Professionals can work on different levels. For example, they can be policy-makers, decision-makers, managers and practitioners. Professionals who practice in areas of ageing and health play an important role in enhancing the older peoples’ possibilities for healthy aging, for example health care professionals, managers or decision-makers. However, it is also important to acknowledge the relevance of people working in areas influencing older peoples’ everyday life, and who have context-specific knowledge that is valuable for the research project, but who are not commonly seen as professionals [13], such as assistant nurses. Hence, in this study professionals are defined as mediators of context-specific knowledge [13]. An argument for involving professionals is that it facilitates implementation by shortening the time required to incorporate research into practice. This might otherwise take a long time [16]. Professionals play an important role in translating knowledge from research to practice and vice versa, thereby facilitating the exchange of knowledge, which is highlighted in the Knowledge Translation framework on ageing and health [17].

Moreover, in response to increasing numbers of older people [18], and a gap between research and practice which means that people do not always receive the best care [19], there is a need for relevant, applicable and sustainable scientific knowledge within the field of ageing and health to ensure greater benefit for the intended beneficiaries of research. One way of obtaining relevant, applicable and sustainable knowledge may be through involving professionals in research on ageing and health.

The involvement of professionals is described as leading to benefits, challenges, obstacles and cost for the researchers, the professionals and the research itself [1, 15, 20–22]. When investigating researchers’ experience of involving professionals, a deductive way of analysis has been used, either by applying a framework [15] or by being informed by literature [1, 20]. Articles describing researchers’ reflections from collaborating with professionals [23] and researchers’ arguments for collaborating with professionals [21, 22, 24] illustrate what the involvement might lead to. A review study found that there is limited objective evaluation of what the involvement of professionals might lead to [25]. However, another review study suggested there was a relationship between professionals’ involvement in research and improved health care processes and outcomes [26].

The growing interest in involving professionals in research has resulted in researchers acquiring more experience in the area, which makes it possible to gain deeper knowledge by involving the researchers in exploring their experiences in the area of ageing and health and developing conceptual areas. There is a need for a clear picture of both researchers’ and professionals’ perspectives to gain a comprehensive insight, though it is also important to differentiate between the two perspectives. This study only focuses on the researchers’ perspective in order to gain a clear and delimited picture of their experiences. Therefore, the aim of this study was to identify conceptual areas of professionals’ involvement in research projects on ageing and health, from the perspective of researchers themselves.

## Method

This study is part of the broader research programme UserAge [27], which focuses on developing knowledge about involving people from outside academia in research on ageing and health, such as frail older people, informal carers and professionals.

Group concept mapping (GCM) was used to capture and conceptualize researchers’ experiences of involving professionals in research on ageing and health. GCM is a mixed method that combines qualitative and quantitative methods and results in a map of conceptual areas [28]. An exploratory sequential approach is employed [29] by means of that the qualitative research phase precedes and informs the quantitative phase [30]. A conceptual area defines a concept and its content. It concretizes the concept by specifying indicators of the content and establishing boundaries of the concept area [31]. GCM is a structured method with a number of steps: a first planning phase, brainstorming, a second planning phase, organizing, analyses, interpretation and use [28]. In the first phase of planning, the project plan is finalized, a focus prompt is developed and relevant participants are recruited. The participants are involved through brainstorming, where they are asked to provide an ending for the focus prompt and thereby generate statements. In the second phase of planning, all the statements which have been generated are reviewed and synthesized. Rating questions are developed so that participants can rate the statements. The organizing step involves the participants being asked to sort the statements into groups and to rate them according to the rating questions by using a predefined scale. The data are then analysed using multidimensional scaling (MDS) analysis and hierarchical cluster analysis. The steps involving interpretation and use of the results are conducted in collaboration with the participants or other relevant users of the research [28]. The Concept System® groupwisdom™ (Concept Systems Inc., Ithaca, NY) has been especially developed to support this method. It can be used in all or some of the steps to support participants’ involvement and to support the researchers in the analysis of the data.

### Procedure and sample

#### First planning phase

During the first planning phase the aim of the study was clarified, and the focus prompt “Involving professionals in research on ageing and health can lead to…” was evaluated during a pilot brainstorming session. Initially, the prompt was phrased using the words “contribute to…”, but it was rephrased to “lead to…” in order to capture both positive and negative aspects. During this phase, relevant participants were identified and invited to take part in the study. Purposeful sampling [32] was used in the search for relevant participants with experience in the topic of interest, and was conducted on a national level through the network of leading researchers within the field of ageing and health, striving for heterogeneity. The inclusion criteria were that participants should have a PhD or be a PhD student, have experience in involving professionals in one or more parts of the research process, and have experience of research in the field of ageing and health, such as health and social care of older people, or rehabilitation and supportive environments. Involvement was defined as “more than just being interviewed or answering a survey”. However, it could imply varying degrees of involvement, from consultation to collaboration.

**Fig. 1:**
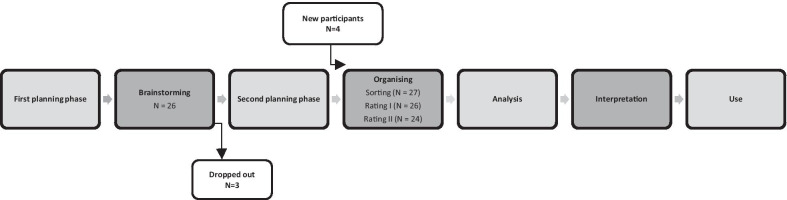
Overview of the group concept mapping process and number of participants (*n*) in each step. The steps that involved the participants are illustrated with darker shading

Potential participants received written and oral information about the aim of the study and the estimated use of time during their involvement. If they accepted, they were asked to complete a letter of consent and a questionnaire on gender, academic level and research experience (Table 1). Participants were also asked to describe in what circumstances they had involved professionals. Most participants had experience of involving professionals in the following ways: as members of a reference group or steering group, or by having them give advice on the research or help to recruit participants, collect or interpret data, or disseminate results. A total of 36 researchers were asked to participate in the study, and six researchers declined to participate, giving time constraints as their main reason. In all, 30 researchers participated in the study (Table 1). The participants received additional information, both written and oral, specifically related to their participation in the different steps. Some researchers participated in all steps of the process, and some only in one or two. For an overview of the GCM process and the number of participants in each step, see Fig. 1. Three participants dropped out after the brainstorming, and four new participants agreed to participate in the organizing phase. There was additional dropout during the organizing phase where some participants did not conduct all the steps.

**Table 1:** Characteristics of participants in the different steps of the GCM process

	Brainstorming	Organizing		
	*n* = 26 (%)	Sorting *n* = 27 (%)	Rating I *n* = 26 (%)	Rating II *n* = 24 (%)
Sex				
Women	20 (76.9)	20 (74.1)	19 (73.1)	17 (70.8)
Academic level				
PhD student	4 (15.4)	4 (14.8)	4 (15.4)	4 (16.7)
PhD	11 (42.3)	11 (40.7)	10 (38.5)	10 (41.7)
Associate professor	5 (19.2)	7 (25.9)	7 (26.9)	5 (20.8)
Professor	6 (23.1)	5 (18.5)	5 (19.2)	5 (20.8)
Research experience, in years				
1–4	3 (11.5)	3 (11.1)	3 (11.5)	3 (12.5)
5–10	8 (30.8)	7 (25.9)	6 (23.1)	6 (25.0)
≥ 11	15 (57.7)	17 (63)	17 (65.4)	15 (62.5)

Rating I: Rates on a scale from 1 (not at all) to 4 (very much) the extent to which the statements can strengthen *research* involving professionals

Rating II: Rates on a scale from 1 (not at all) to 4 (very much) the extent to which the statements can strengthen *practice* in research involving professionals

#### Brainstorming

To facilitate the involvement of the participants, several brainstorming sessions (between November 2018 and April 2019) were conducted on different days and in different places. A total of 26 people participated in these brainstorming sessions, which were divided into six group sessions with two to six participants in each. The participants were offered the choice of participating face to face or online (by using video conferencing platforms) and could choose what suited them best. Three of the sessions were face to face meetings, in two sessions a combination of face to face and online participation was used and one session was solely online. Two participants brainstormed individually by using the web-based Concept System® groupwisdom™. Before the brainstorming session, participants were given instructions to complete the focus prompt according to their experience and knowledge. During brainstorming they were encouraged to think about both positive and negative aspects of what involving professionals can lead to in relation to research and practice. The rules for brainstorming were followed [28]; for example, participants were asked to brainstorm freely and not debate the statements, since the aim was to capture their broad experiences and knowledge, not to reach consensus. Moreover, participants were asked to restrict each statement to one focus only, otherwise it would have to be split into two statements. Two of the authors participated in each session, one facilitating the brainstorming and one writing down the statements, which were displayed directly on a screen for all participants.

#### Second planning phase

During the second planning phase, all statements from the brainstorming sessions were reviewed and synthesized. The reason for synthesizing is to have a manageable number of statements for the participants to sort and rate, and at the same time to ensure that the statements are representative as well as saturation of the participants’ experiences of the topic [28, 33]. During the process of reviewing and synthesizing, the first author marked statements not related to the focus prompt, which upon confirmation from the other authors were removed. The first author sorted statements with the same or a similar meaning, or keywords, into a horizontal row in a document. This helped find the statement that best captured the meaning of the statements in each row and enabled an audit trail of the process. The list of statements was reviewed by the second author, and then discussed by all the authors together and reviewed further, resulting in a final list of statements. To enable confirmability [34] all the authors could keep track of the synthesizing process since all statements were viewed in the document during the process.

#### Organizing

Later in the organizing step, participants individually sorted (*n* = 27) and rated (rating I, *n* = 26; rating II, *n* = 24) the statements in the web-based Concept System® groupwisdom™. By using the web-based system the participants were able to conduct the sorting and rating when they wanted, at their own pace within a time frame of 10 weeks. Three of the 26 participants who participated in the brainstorming session did not conduct the sorting and rating. However, four other participants who were interested in participating in the study but were unable to participate in any brainstorming sessions chose to participate in only the organizing step. The GCM method is flexible in that way; it is not necessary for the same people to participate in all the steps [35].

In order to conduct the sorting, the participants were asked to sort the statements into groups on the basis of how they perceived that the statements related to each other, and to label each group. The participants were informed that it was not acceptable to sort all the statements into one group, and that they should only place a statement on its own in a group if it was in no way related to any other statement. Furthermore, participants were instructed that they should not create groups according to priority or value, such as “important” or “difficult to do”, and that they should avoid creating groups which covered a variety of statements, such as “miscellaneous” or “other”.

Next, the participants were asked to rate all statements according to the following two questions: “To what extent can the following statements strengthen *research* conducted with the involvement of professionals?” (rating I) and “To what extent can the following statements strengthen *practice* when conducting research with the involvement of professionals?” (rating II). *Strengthening* was defined for the participants as developing relevant, applicable and/or sustainable knowledge. The participants were instructed to use a scale from 1 to 4 (1 = not at all, 2 = a little, 3 = a lot, 4 = very much), and to use the entire range of response categories.

### Analyses

MDS analysis and hierarchical cluster analysis were conducted using the Concept System® groupwisdom™. Calculating a similarity matrix, the MDS analysis, based on how the participants sorted the statements into groups, resulted in a point map where each point represents one statement. The point map is a two-dimensional map that illustrates the relations between the statements as a result of how these have been sorted by the participants. Statements which were most frequently sorted together appeared closer to each other on the point map [36]. A low stress value represents a better fit between the similarity matrix for the MDS and the point map [33], and in GCM studies the stress value often lies between 0.10 and 0.35 [28]. Rosas and Kane [33] estimate that acceptable stress values can be achieved with a sample of 20–30 participants.

Following the MDS analysis, a hierarchical cluster analysis calculated the cluster solution. Bridging values (BVs) were calculated for each statement, as well as the average BV for each cluster. BV ranges from 0 to 1 and describes an aggregation of the sorting by all participants. The values refer to how the statements are related to other statements. The lower the BV, the more the statement is anchored to its place on the map, meaning that it has been sorted with statements that are in the same area on the map. The higher the BV, the more a statement has been sorted with statements placed further away on the map, thereby bridging to other areas on the map. Statements with a low BV are more representative of the meaning of the cluster in which they are located than those with higher BVs. The BV for a cluster is the average of all the BVs for the statements in each cluster. Lower BVs represent a more homogeneous cluster and higher BVs a more heterogeneous cluster [28].

The first and second author discussed labelling of the clusters. They read through all the statements in each cluster and interpreted the content of the cluster in a qualitative way, at the same time looking both at the participants’ suggestions for labels as well as statements with a low BV, i.e. representing “anchors” within that cluster. The label decided on encapsulated the conceptual content of the cluster. GCM maps include observable features, which are descriptive and manifest, as well as hypothetical, unobservable features, which are latent [31]. Thus, a qualitative analysis [37] of the latent meaning of the cluster map was then carried out inductively, by grouping clusters into two themes based on the content of the clusters.

The data from the ratings of the extent to which the statements could strengthen research (rating I) and practice (rating II) from 1 (not at all) to 4 (very much) were then analysed by plotting the statements in a bivariate scatterplot, known as a go-zone map in GCM studies. The go-zone shows the mean rating of each statement, thereby illustrating similarities and differences in how the participants rated the statements [28]. The go-zone map was used to analyse and illustrate associations between the two ratings (rating I and rating II) of the statements.

#### Interpretation and use

The possibility to conduct member check to validate the interpretation of the results [34] was given through a seminar when the authors met other researchers who had experience of involving professionals in research, and some of the participants who had taken part in the study. The preliminary maps were presented, and to check the validity of the authors’ interpretation of the clusters they were especially asked to verify the labels of the clusters in relation to the statements. No changes to the results were suggested.

According to Rosas [31], a GCM map is the basis for developing an explanation for and expanding understanding of the area under investigation, since “conceptual models generated in concept mapping are devices to help individuals and groups understand the real world more clearly” (p.1413). GCM is often used as a method for evaluating or planning interventions, and developing instruments or theories [28]. The discussion and conclusion will illustrate how the results from this study can be used.

### Ethical considerations

Before accepting an invitation to take part, participants received written and oral information about the project and about what they could expect if they participated, and they were informed that all data would be handled confidentially. Participation was voluntary, and they were informed that they could end their participation at any time. They were informed that the brainstorming sessions would be in groups, meaning that they would not be anonymous. However, the organizing phase would be carried out individually, and the results would be presented on group level without any possibility to identify individual persons. Furthermore, they had the opportunity to contact the researchers with any questions or problems related to their involvement in the web-based parts of the study.

## Results

A total of 512 statements were generated during the brainstorming phase. The statements were reviewed and synthesized, resulting in a final list of 94 statements. Table 2 lists the statements within the five clusters, the BVs of clusters and statements, and the mean ratings of the extent to which the statements could strengthen research (rating I) and practice (rating II).

**Table 2:** Ninety-four statements of what professionals’ involvement in research on ageing and health can lead to, within five clusters

Cluster solution and statements		Bridging value^a^	Rating I research^b^	Rating II practice^c^
Cluster 1: Complex collaboration throughout the research process		*0.25*	*2.30*	*2.09*
1	Ethical challenges	0.32	2.52	2.50
3	Special considerations to be taken regarding confidentiality	0.12	2.68	2.08
4	Research projects taking a long time	0.02*	2.48	2.13
5	A demand for greater engagement from the researcher	0.49	3.04	2.61
6	Decision-making processes in research affected	0.22	2.69	2.17
7	Employees question the professionals’ priorities of work tasks	1	1.88	2.09
8	More complex and demanding role of the researcher	0.58	2.92	2.10
9	Unclear roles for the researchers	0.16	1.71	1.75
10	Uncritical approaches from the researchers	0.15	1.80	1.52
12	Dilemma for the researchers if they need to make demands on the professionals	0.2	2.28	1.75
14	Demands on the researcher to make the research understandable and accessible	0.81	3.64*	3.58*
16	That researchers must protect their integrity	0.43	2.08	1.87
17	Frustration for the researcher in safeguarding time and quality	0.05	2.12	1.63
20	Situations where researchers need to mediate and negotiate	0.04	2.31	2.00
21	Difficulties for the researcher in deciding how much the professional should be involved in interpreting results	0.02*	2.00	1.91
23	Heavy demands on professionals’ engagement	0.43	2.56	2.83
32	Professionals focusing too much on their own work environment	0.15	1.84	1.92
33	Professionals feeling questioned	0.13	1.85	1.88
52	Research that demands more resources	0.08	2.67	2.09
53	In grant applications, challenges in describing how professionals should be involved	0.2	2.52	2.17
57	Diminishing research freedom	0.04	1.72	1.50
58	An unpredictable research process	0.07	2.52	2.25
68	Difficulties in establishing a research group with the right skills due to unpredictable processes	0.09	2.08	1.78
70	Difficulties in arranging meetings and continuity between participants (professionals)	0.07	2.36	2.26
74	Conflicts between professionals and researchers	0.12	1.96	2.00
79	Unclear roles for professionals	0.32	1.77	1.87
87	Crisis of confidence where one part feels like a “hostage”	0.12	1.63	1.57
88	Increased demand for clarifying roles and frameworks for the project	0.52	2.92	2.61
Cluster 2: Adaptation of research to different stakeholders		*0.3*	*2.06*	*2.01*
11	Time-consuming collaboration to create equal relationships	0.29	2.40	2.25
13	Increased demands for creativity and flexibility from the researcher	0.4	3.20*	2.91
34	Ownership for professionals	0.37	2.40	3.17*
42	Less generalizable knowledge	0.47	1.68	1.63
55	The research community questioning the quality of the research	0.22	2.42	2.00
59	Populist research	0.41	1.60	1.68
64	Compromising the scientific quality	0.3	1.56	1.38
69	Risk of commissioned research expected to be driven in a given direction	0.08	2.08	2.17
71	Poor research	0.2	1.60	1.42
76	Power balance between researchers and professionals	0.54	2.44	2.50
77	Non-democratic processes between researchers and professionals	0.32	1.65	1.54
83	Conflicts of interest between researchers and professionals	0.25	1.96	1.79
84	Distortion of research results caused by the professionals’ own interests	0.07*	1.84	1.70
Cluster 3: Mutual learning through partnership		*0.41*	*3.20*	*3.24*
22	Researchers gaining greater understanding of professionals’ perspectives	0.47	3.44	3.33
25	Researchers and professionals identify with each other	0.75	2.36	2.38
30	Professionals and researchers inspiring each other	0.41	3.64*	3.42
31	Heavy demands on communication between researchers and professionals	0.74	3.44	3.26
39	Facilitation of communication	0.29	3.00	3.13
40	Increased collaboration in new projects	0.38	3.04	2.71
56	Increased transparency	0.31	3.20	3.17
65	Good relations between academia and practice	0.23	3.16	3.22
66	Collaboration where researchers and professionals work towards common goals	0.53	3.24	3.54
75	Mutual learning	0.2*	3.24	3.63*
78	Increased understanding and knowledge of each other’s area of expertise	0.27	3.32	3.29
80	Respect for each other’s knowledge	0.48	3.12	3.38
81	Researchers and professionals feel mutual responsibility	0.43	3.16	3.33
82	A partnership in developing health care	0.26	3.48	3.57
Cluster 4: Applicable and sustainable knowledge		*0.13*	*3.18*	*3.22*
19	Real change in society	0.02	2.92	2.78
24	Increased legitimacy of research results among professionals involved	0.08	3.36	3.48
26	Professionals mediating research results	0.09	2.88	3.43
28	Professionals enabling research	0.22	3.25	3.21
35	Professionals gaining increased understanding of research	0.26	3.04	3.38
37	Practical development of the organization	0.05	2.92	3.46
38	Development of new methods for involving professionals	0.13	3.28	2.96
41	Efficient use of resources	0.11	2.64	2.75
43	A holistic perspective	0.14	3.32	3.33
44	Critical views emerge	0.22	3.32	2.88
45	Contextually adapted relevant research	0.1	3.62*	3.48
46	The identification of future needs	0.1	3.32	3.35
47	Increased sustainability of results and working methods	0*	3.12	3.43
48	More complex knowledge acquired through dialogue	0.19	3.48	3.29
50	Increased access to data	0.09	3.21	2.91
51	Increased scientific quality in the research	0.16	3.32	3.04
60	Changed attitudes to and understanding of research	0.06	3.13	3.35
61	Narrowing the gap between research and practice	0.13	3.36	3.46
62	Impact on guidelines and procedures in practice	0.12	2.71	3.04
63	Increased collaboration between authorities	0.22	2.88	3.08
67	Facilitated implementation of research results	0.07	3.31	3.50
72	Increased knowledge of different organizations’ conditions	0.15	3.16	2.88
85	Reliable results	0.19	3.28	3.42
86	Facilitation of knowledge dissemination	0*	3.36	3.57*
89	New networks for researchers and professionals	0.23	3.24	3.00
Cluster 5: Legitimate research on ageing and health		*0.57*	*2.94*	*3.03*
2	Ethically justifiable research	0.76	3.28	3.13
15	Researchers perceiving research conducted as more meaningful	0.8	3.50*	2.74
18	Researcher acquiring more competencies	0.76	3.29	2.58
27	Professionals are heard and seen as experts	0.62	3.08	3.33
29	Professionals grow in their roles	0.47	2.80	3.50*
36	A more attractive workplace for professionals	0.66	2.44	3.17
49	Important research questions that the researcher was unaware of	0.61	3.36	3.04
54	Granting of applications for grants	0.94	3.00	2.38
73	Organizational conditions are changing	0.5	2.30	2.70
90	An impact on age discrimination	0.96	2.04	2.57
91	Increased knowledge about older people’s needs	0.22	3.24	3.39
92	Better health care for older people	0.17*	3.04	3.45
93	Greater degree of person centredness in practice and research	0.32	2.76	3.21
94	Increased understanding of ageing	0.26	2.96	3.26

^a^Bridging value: The mean value for all the bridging values of statements within the cluster is shown in italics. An asterisk (*) shows statements with the lowest bridging value within a cluster

^b^Rating I: Research: Mean rating on a scale from 1 (not at all) to 4 (very much), from the rating of the extent to which the statements can strengthen research. The mean rating for all the statements within the cluster is shown in italics. An asterisk (*) shows statements with the highest rating value within the cluster

^c^Rating II: Practice: Mean rating on a scale from 1 (not at all) to 4 (very much) from the rating of the extent to which the statements can strengthen practice. The mean rating for all the statements within the cluster is shown in italics. An asterisk (*) shows statements with the highest rating value within the cluster

**Fig. 2:**
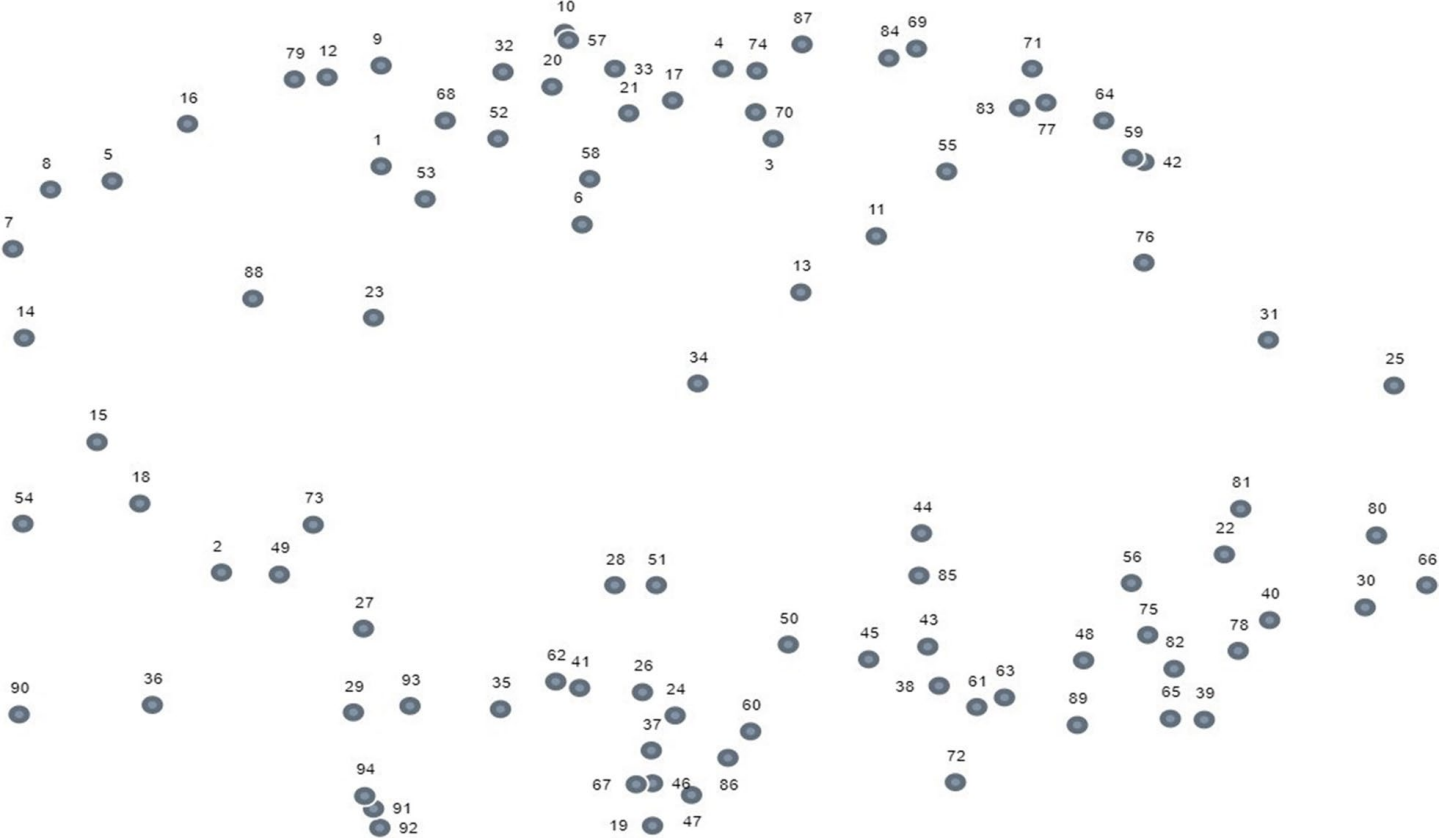
Point map

An MDS of the sorted statements resulted in a point map (Fig. 2). The stress value of 0.26 for the point map indicates a good fit between the raw data and the result. The point map illustrates how the statements are related. Statements which are sorted together more often are placed near each other; therefore, it is the relationships between the points that are of interest.

**Fig. 3:**
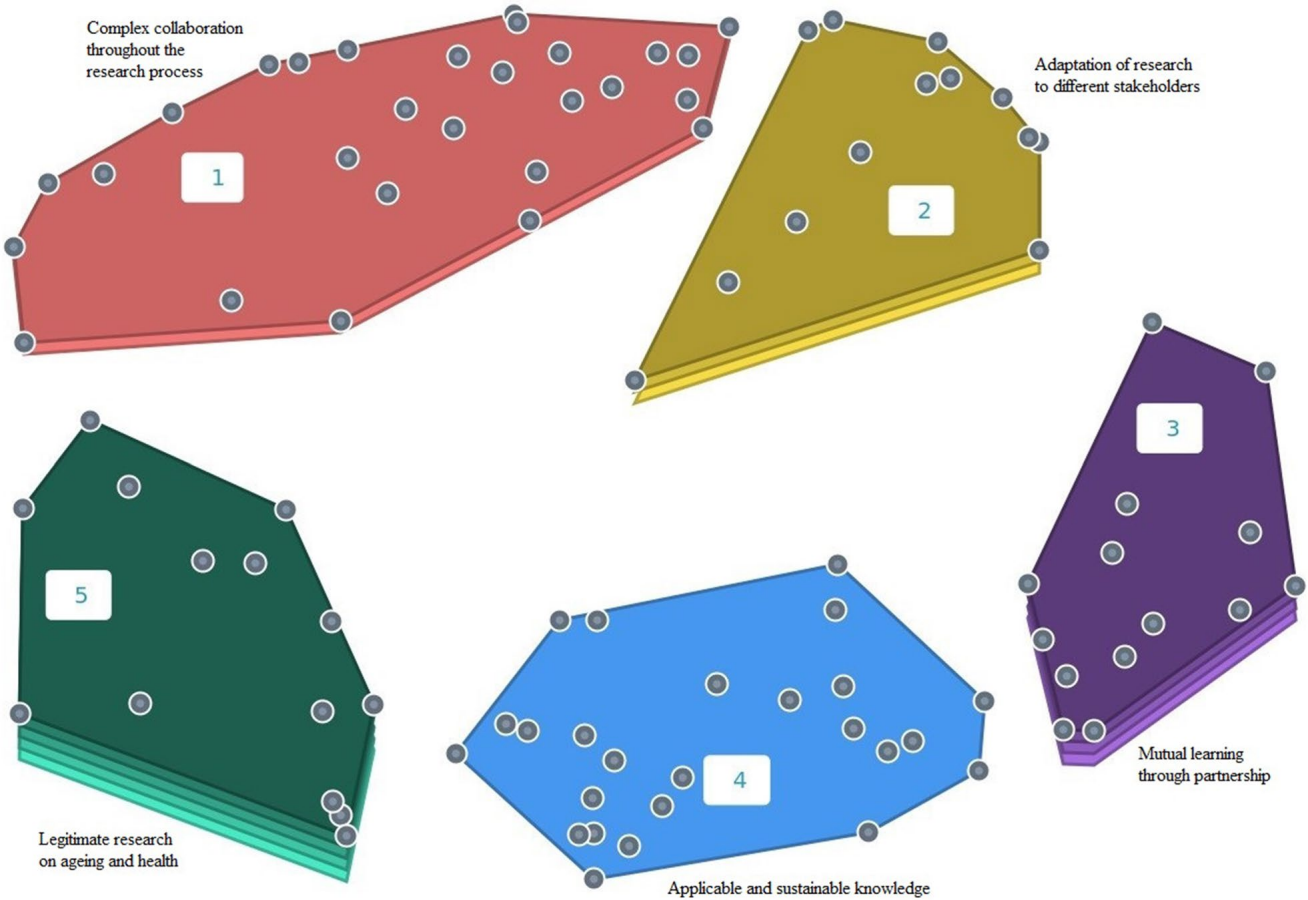
Point cluster map

Hierarchical cluster analysis was used to create a map of conceptual areas, and a solution with five clusters was chosen (Fig. 3) because of the coherency of the statements in the clusters. BVs for the clusters and statements were also taken into consideration in deciding on the cluster solution.

The five clusters are as follows: complex collaboration throughout the research process (cluster 1); adaptation of research to different stakeholders (cluster 2); mutual learning through partnership (cluster 3); applicable and sustainable knowledge (cluster 4) and legitimate research on ageing and health (cluster 5). The BVs of the clusters are illustrated as layers in the clusters; the more layers there are in a cluster, the higher the BV of the cluster (see Fig. 3).

Cluster 1 (complex collaboration throughout the research process) has an average BV of 0.25, indicating that it is a homogeneous cluster. Statement 4, “research project taking a long time”, and statement 21, “difficulties for the researcher in deciding how much the professional should be involved in interpreting results”, are the most anchored statements in the cluster with the lowest BVs (0.02). The cluster illustrates the complex collaboration between researchers and professionals involving challenges regarding aspects of roles and responsibilities in research. Cluster 2 (Adaptation of research to different stakeholders) has an average BV of 0.3. Statement 84, “distortion of research results caused by the professionals’ own interests” is the anchor for this cluster (BV = 0.07). This cluster illustrates aspects of the process when research is adapted in order to involve professionals. It demonstrates the need for balance between aspects valued by research and aspects valued by practice which can result in conflicts but also in a feeling of ownership for professionals. Cluster 3 (mutual learning through partnership) has an average BV of 0.41, and statement number 75, “mutual learning” (BV = 0.2), has the lowest BV. Learning is described in this cluster as achieved by both the researchers and the professionals. They are inspired by each other and gain an understanding of each other. Respect, communication and good relationships foster the partnership. Cluster 4 (applicable and sustainable knowledge) has an average BV of 0.13. Two of the statements have a BV of 0: statement 47, “increased sustainability of results and working methods”, and statement 86, “facilitation of knowledge dissemination”. This cluster illustrates aspects of what applicable and sustainable knowledge can achieve. The professionals’ role in mediating and enabling context-relevant knowledge is highlighted as a facilitating element. Cluster 5 (legitimate research on ageing and health) has an average BV of 0.57, and is therefore the most heterogeneous cluster. Statement 92, “better health care for older people” has the lowest BV (0.17), and is therefore the anchor statement in this cluster. This cluster illustrates aspects of how legitimacy is achieved for research on ageing and health. Considering the professionals as experts, and seeing ethical justifiable research and outcomes that benefit the researchers, the professionals as well as the older people are factors contributing to legitimacy.

**Fig. 4:**
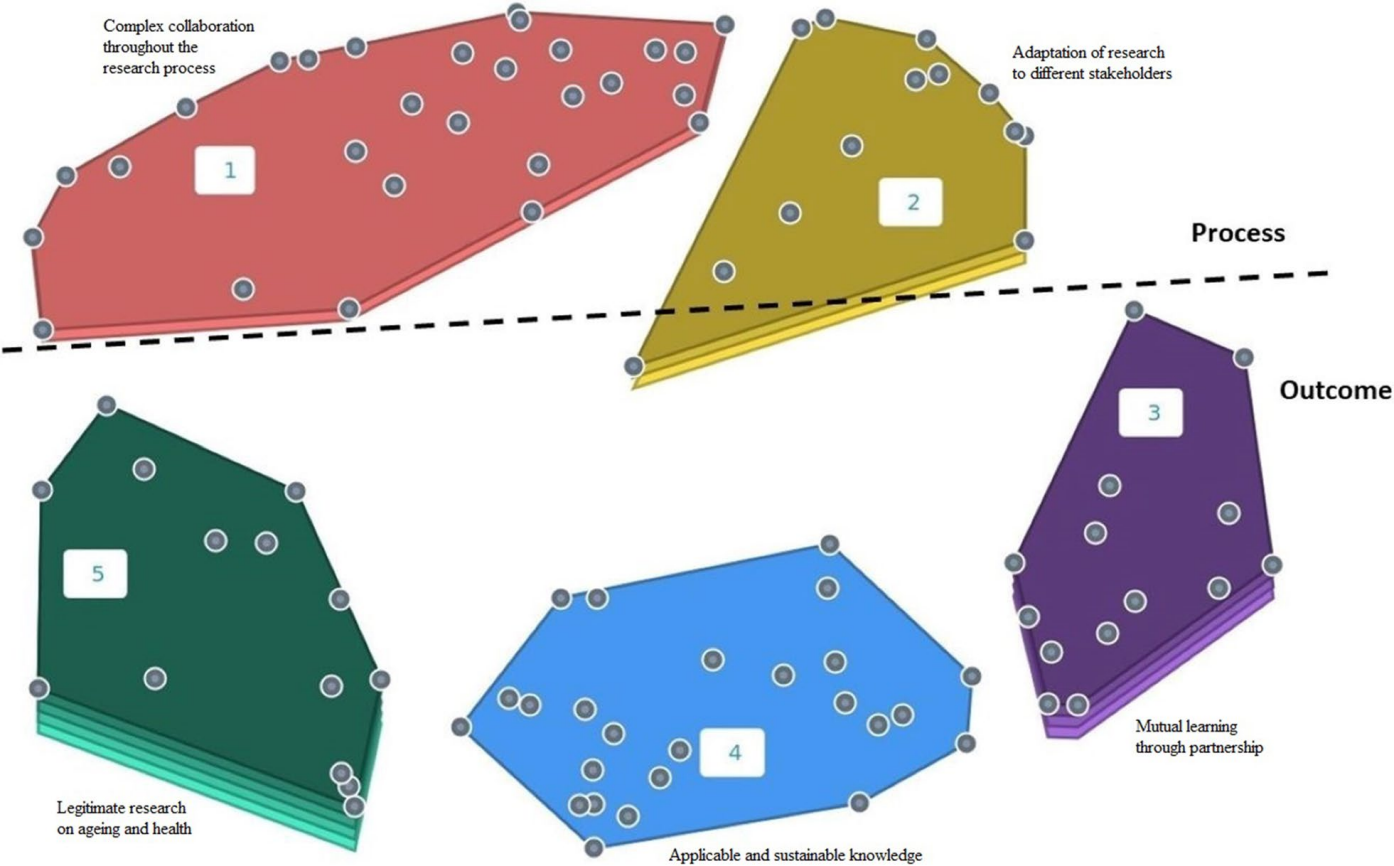
Point cluster map; qualitative analysis of the latent meaning of the conceptual areas

Through further qualitative analysis of the latent meaning of areas, the two clusters at the top of the map, cluster 1 (complex collaboration throughout the research process) and cluster 2 (adaptation of research to different stakeholders), can be seen as comprising conceptual areas related to the *process* of involving professionals in research on ageing and health, whereas the three conceptual areas at the bottom, cluster 3 (mutual learning through partnership), cluster 4 (applicable and sustainable knowledge) and cluster 5 (legitimate research on ageing and health), can be seen as related to the *outcome* of involving professionals in research (Fig. 4).

The mean rating of the five clusters (see Table 2), or the extent to which they strengthen research and practice, ranges from 2.01 to 3.24, indicating that most of the statements were rated high on the scale from 1 to 4. Cluster 3 (mutual learning through partnership) was rated highest in terms of both strengthening research (3.20) and strengthening practice (3.24). Cluster 2 (adaptation of research to different stakeholders) was rated lowest in terms of both strengthening research (2.06) and strengthening practice (2.01).

**Fig. 5:**
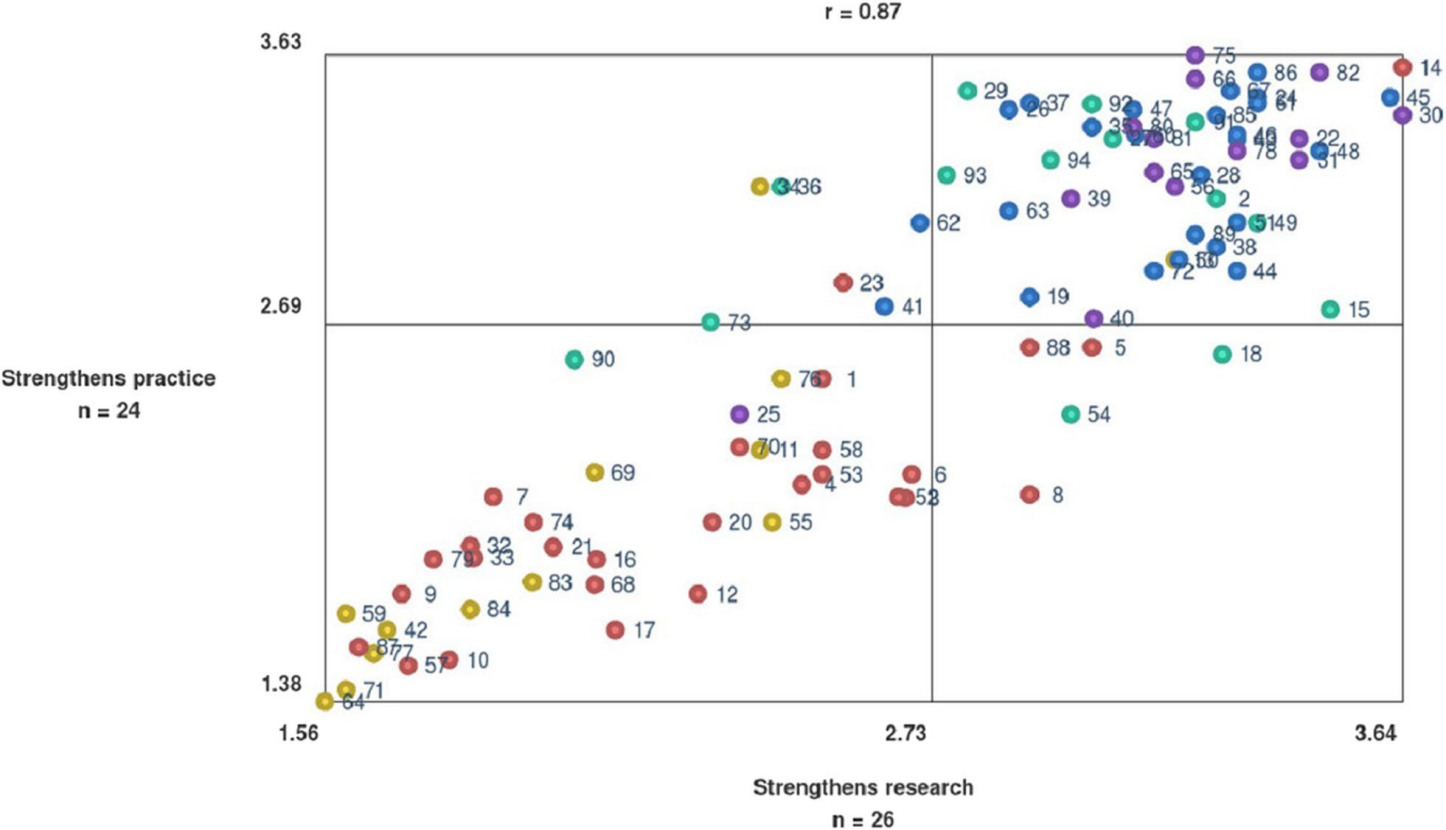
Go-zone map. The go-zone map shows the mean of the participants’ ratings for each statement. *X*-axis (strengthens research), *y*-axis (strengthens practice). The colours of each point illustrate which cluster the statements belong to (see Fig. 3)

The go-zone map (Fig. 5) is a bivariate scatterplot where the statements are visually placed on an *x*-axis (strengthens research) and a *y*-axis (strengthens practice), according to the mean of the participants’ ratings for each statement. There is a strong positive correlation (0.87) between the statements rating: the more a statement is graded to strengthen research, the more it is graded to strengthen practice, and vice versa.

The go-zone map is divided into quadrants above and below the mean of all statement ratings in terms of strengthening research and practice. The mean for strengthening research is 2.73 and the mean for strengthening practice is 2.69. The statements in the upper-right quadrant, the go-zone, are rated high in terms of strengthening both research and practice, and are mostly statements from clusters related to outcome: mutual learning through partnership (cluster 3), applicable and sustainable knowledge (cluster 4) and legitimate research on ageing and health (cluster 5). The lower-left quadrant, the “o-zone”, contains statements which are rated low in terms of strengthening both research and practice. On this map, the o-zone mostly contains statements from clusters related to the process: complex collaboration throughout the research process (cluster 1) and adaptation of research to different stakeholders (cluster 2). The two other zones, the gap zones, contain statements which are rated high in one and low in the other for research or practice.

## Discussion

The aim of this study was to identify conceptual areas of professionals’ involvement in research on ageing and health, from the perspective of researchers themselves. Using the GCM method enabled the study to capture the range and diversity of the participants’ knowledge and experience in terms of involving professionals in research on ageing and health, and to merge it into one map of conceptual areas. The following discussion of the results is based on a qualitative analysis of the latent meaning of the cluster map, *the outcome of involvement* and *the process of involvement*. The results of this study illustrating conceptual areas emphasize the need for a holistic view including both the process and the outcome when involving professionals. Other studies on research partnership also emphasize the relationship between process and outcome. For example, Tabriz et al. [38] structured their findings in terms of inputs to, processes of and outcomes from the partnership, and Nyström et al. [1] illustrate the research partnership process as preparation, process and impact. Furthermore, the results of this study also contribute by illustrating what the involvement can lead to during the process as well as by giving a more extensive description of the outcomes.

### Outcome of involvement

The conceptual areas “mutual learning through partnership”, “applicable and sustainable knowledge” and “legitimate research on ageing and health” can be seen as related to the outcome of involving professionals in research projects on ageing and health. The participants’ experience that involving professionals leads to mutual learning through partnership illustrates that a development occurs not only for the professionals involved and their practice but also for the researchers and their research. This is illustrated in the statements “professionals and researchers inspiring each other” and “the researcher gaining greater understanding of the professionals’ perspectives”. This is in line with the results of Staley’s [39] narrative literature review which is conducted within the context of patient and public involvement. The findings show that researchers learn by acquiring new knowledge and communication skills through patient and public involvement. Also, Staley’s [39] study found that the researchers’ attitudes to involvement change, which might ultimately change how they conduct their research. Mutual learning is described as an outcome of participatory research when the researcher and the people involved work together as colleagues [40], and is argued to be important for the process in research partnerships with professionals [41].

Our finding that mutual learning takes place through partnership aligns with the assumption that people learn through social relationships, or in other words that people can co-create new knowledge by sharing constructions of their reality or worldview [42]. In turn, it is more likely that the co-created knowledge will fit the context of practice, and thereby be more relevant and applicable. This is exemplified in the conceptual area illustrating that involvement of professionals leads to applicable and sustainable knowledge. Hence, applicable and sustainable knowledge cannot be created isolated from its context [42], in this case practice, since it is historically and socially constructed [43]. This emphasizes the importance of involving the professionals who can benefit from the research, and developing mutual learning through collaboration. According to Lincoln and Guba [42], the way people see and understand realities develops when they learn from others by gaining new knowledge, which enables them to adapt their understanding of the world. If the new knowledge they gain adds to their understanding of the world, their aggregated knowledge is more likely to become applicable and sustainable, generating “contextually adapted, relevant research”, as one of the statements in the study suggested. Sustainable knowledge which leads to an impact on society is considered to be an outcome of the collaborative process in a number of co-creation models, where stakeholders are involved [44]. Furthermore, applicable knowledge being more context-sensitive is highlighted as a means of enabling implementation of the knowledge [17, 24].

Involving professionals in a collaborative process has an underlying democratic ideology which aims to legitimize research on ageing and health. Accordingly, the results of the present study show that legitimate research on ageing and health is a conceptual area illustrating what involvement of professionals can lead to. Legitimacy can be achieved when the research process is viewed as fair and ethical. This entails respect of different values and interest in an unbiased and fair process by genuine inclusion of stakeholders in the process of collaboration [45]. Thereby legitimacy may depend on how decisions are made and whether they are widely accepted by people. The statement “professionals are heard and seen as experts” confirms that professionals can influence research and can have a say by being involved in it, and this, in turn, contributes to the legitimacy of the research. However, in itself, research is often legitimized through standardized and precise rules or proof for how “something” works, which is in keeping with the “legal-bureaucratic model” described by Rothstein [46]. Rothstein [46] describes several models to explain how policies for the welfare state are legitimized in a democratic way. The “legal-bureaucratic model” and the “professional model” are of particular interest in terms of the legitimacy of research on ageing and health. In the “legal-bureaucratic model”, governance and decisions are built on precise, standardized rules, where people are acquainted with the regulations and can predict the outcomes. In the “professional model”, legitimacy is the result of leaving licensed professionals to decide how to implement decisions. Licensed professionals have specific knowledge and ethics which they have acquired through an established education, so they can be designated the responsibility for making decisions in their work [46]. When professionals are involved in research, they represent their organization, and bring specific knowledge and ethics which enable them to help adapt research to the context, that is, the practice. This is illustrated in the statement “greater degree of person centredness in practice and research”. This is well in line with a study on transdisciplinary research (collaboration between researchers and different professionals) in sustainable urban development which showed that legitimacy could be achieved by involving different expertise and perspectives in the process [47].

The conceptual areas interpreted as outcomes were rated high on the go-zone map in terms of strengthening research and practice. Interestingly, there was a strong positive correlation (0.87) between statements rating. Statements and clusters in the upper-right quadrant of a go-zone map are often those most emphasized in projects for evaluation or development [48, 49] since they are rated highest and are often rated according to importance and feasibility. However, it is important to reflect on the clusters and statements in the o-zone and gap zones as well, as they may have a more philosophical foundation. In addition, all clusters and statements are related to each other, as illustrated by the BVs. The go-zone (Fig. 5) shows that the conceptual areas related to the process of involving professionals are rated low in terms of strengthening research and practice. However, it could be argued that it is not possible to reach the conceptual areas related to outcome without successfully “going through” the conceptual areas related to the process, since involving professionals in research is not a linear process. The conceptual areas related to the process may not direct or clearly strengthen research or practice but by looking at the content of these areas it can be argued that they are equally important. Hence researchers’ considerations of what the involvement of professionals may lead to should be supported by considerations of how to involve. Drawing links between process and outcome and investigating the relationship is important since there is limited knowledge of what in the process of involvement might lead to different outcomes given the complexity of the process and different contexts [25, 26].

### Process of involvement

The conceptual areas related to the process of involvement are “complex collaboration throughout the research process” and “adaptation of research to different stakeholders”.

In this study, complex collaboration throughout the research process is illustrated by several statements related to aspects of the roles and responsibilities of researchers and professionals. One of the statements indicates that involving professionals leads to an “increased demand for clarifying roles and frameworks for the project”. Unclear definitions of roles and expectations are shown to be a barrier to collaboration [50]. Role descriptions for those involved in research [51] were suggested as a way of minimizing barriers related to roles. However, involving professionals is a complex and constantly changing process of collaboration, so it is difficult to build on static roles and responsibilities. This can lead to “conflicts between professionals and researchers” and a “more complex and demanding role of the researcher”, as they need to balance the “increased demand for clarifying roles and frameworks for the project” as well as “ethical challenges”. Olswang and Goldstein [22] describe that professionals and researchers have different roles when they collaborate in a research project. They have different knowledge and expertise, and understanding and appreciating this will make collaboration more productive. The statement in this cluster that was rated highest in terms of strengthening research and practice was “demands on the researcher to make the research understandable and accessible”. This illustrates a need to clarify the research process for the professionals involved.

The other conceptual area related to the process of involvement is “adaptation of research to different stakeholders”. When researchers involve professionals, they adapt themselves in order to reach a balance of power, as illustrated in the statements “power balance between researchers and professionals”. This requires qualities from the researchers which are not always explicit. The statement “increased demands for creativity and flexibility from the researcher” provides an example of these qualities. There nevertheless remains a risk of conflict or tension between researchers and professionals, especially when the interests of one party dominate. Several statements in this cluster include examples of these risks, such as populist research, distortion of research results and non-democratic processes. The relationship between researchers and professionals is not without tension, which is described in a study by Bartunek and Rynes [52], where logic, the time dimension and communication illustrate some of the sources of tension. Interestingly, the authors suggest harnessing these tensions to foster change [52]. The theory for expansive learning by Engeström [43] also stresses that “contradictions are the prime source of change” (p.16) in collaboration between different professions. This can be applied to the relationship between researchers and professionals in collaborative research projects. Lofman et al. [53] point out that researchers in participatory action research must be aware of the power balance during the whole process of collaboration, and that professionals must be seen as equals in order to feel a sense of ownership of the project. The statement “ownership for professionals” was rated high in terms of strengthening practice in conducting research where professionals are involved. Furthermore, the statement is located in the centre of the point map (Fig. 2), illustrating its central conceptual relationship with all the other statements. A power balance and a feeling of ownership are linked to the relationship between researchers and professionals, indicating that the way in which researchers encounter professionals in the process of involvement is an essential aspect of the process.

The approach and epistemological considerations of researchers have an impact on how they involve professionals, such as how they encounter the people they involve, which affects the process of involvement [54]. The increased focus on involving people, such as professionals, in research indicates that the definition of scientific evidence has developed to include a broader perspective on what counts as knowledge, and an acceptance of different kinds of knowledge. Building on Polanyi’s (1958) ideas, McHugh and Walker [55] assert that the justification of different kinds of knowledge is related to epistemologies, and they emphasize that tacit, explicit, particular and general forms of knowledge are required in a dynamic combination in research and in health care. However, the statement “the research community questioning the quality of the research” illustrates that, although some researchers value collaboration with professionals, there may be some from other research traditions who do not, since knowledge gained through collaboration is not always acceptable in other research paradigms [56]. Researchers are often schooled in a certain scientific philosophy in terms of what they consider knowledge to be [57], and these views rely on socially and culturally acceptable circumstances in their area and community of research. Kuhn [56] and Guba and Lincoln [57] explain this as researchers working within different paradigms. Therefore, when researchers on the same project have different views on knowledge, it can cause tensions in the research process [54].

This study shows that involving professionals in research can lead to mutual learning, applicable and sustainable knowledge, and legitimate research. However, it is necessary to navigate successfully through the complex research process and adapt to the professionals involved. In this sense, it is crucial that researchers who involve professionals reflect on their underpinning epistemic beliefs in a critical way during the research process and reflect on how these affect not only their views about knowledge but also how they involve professionals in the research process.

### Methodological considerations

By using a mixed method, the strengths of both the qualitative and quantitative analysis give breadth and depth to the understanding of the research question [30]. Through participants’ brainstorming and sorting of statements depending on how they experience that statements relate to each other (qualitative) and the subsequent statistical analysis (quantitative), of the participants sorting and rating, GCM can be considered an exploratory sequential approach [29]. In addition, what is found quantitatively, by means of clusters, is then qualitatively analysed by its latent content. The design of GCM aligns with an exploratory sequential approach; however, as Rosas [58] argues, the method integrates data and analysis at multiple points of the process, thereby intertwining the qualitative and quantitative methods in a more complementary and additive manner, which possibly can be regarded as slightly different from letting the quantitative phase build on the qualitative research phase. Mixed methods use multiple worldviews [32], and GCM was chosen as it enabled the authors to combine a participatory worldview, emphasizing collaboration, with a pragmatic worldview, which focuses on practice and the participants’ real-world issues. The statements were brainstormed by the participants in order to collect their experiences as a basis for the analysis. Predetermined statements can be used in GCM studies and can be defined, for example, from theory or literature [59]. However, a valuable aspect of this study was that the participants were involved and that the statements originated from their own experiences. Participating in a GCM study was believed to be meaningful for the participants per se, but the illustrative and usable results are also considered meaningful for practice and research. GCM is a reliable and valid method [33], but precautions need to be taken to ensure the validity of the qualitative and quantitative data [30], especially given the participatory approach. It was therefore important to make sure that the participants understood the questions and instructions in the steps they were involved in. Our experience is that the participants understood the instructions and questions, and had a greater understanding of the study, precisely because they were researchers who had considerable experience and knowledge of involving professionals in research on ageing and health. This also meant that they could provide rich and complex data. The qualitative data collection conducted through brainstorming sessions generated 512 statements which were reviewed and synthesized, resulting in a final list of 94 statements, indicating that a high degree of saturation of the topic had been reached. To enhance the trustworthiness, all the authors participated in the qualitative analysis. Several of the authors were skilled in both qualitative research and the GCM method, ensuring the enquired sensitivity to the study’s data and process [37]. GCM has been shown to give structure and order to rich and complex data, and thereby enable further interpretation for evaluation, development or decision-making [60, 61]. A concept map constructed through GCM visualizes empirical co-constructions of the participants’ real world [28]. This means that the participants who were researchers themselves co-constructed the map in this study, giving structure to the complex and real-world issues introduced by involving professionals in research.

In a GCM study, participants’ involvement in some of the steps of the research process is rather time consuming, and in this case it resulted in some participants withdrawing due to lack of time. There were a few participants who dropped out after the brainstorming phase and others participated only in the organizing phase. GCM is a flexible method [35] that allows different participants to take part in the different steps, since brainstorming and organizing constitute two completely different elements of the research process. In considering involvement of professionals in research, the study covers areas of involvement extending from consultation to collaboration, and does not differentiate between different approaches of involvement. This may limit the extent to which the result can be used. The conceptual areas of this study focus on professionals’ involvement in research projects on ageing and health; however, the results are believed to be transferable to other research areas given the broad concepts covered in the findings. This study solely includes the researcher’s perspective and thereby limits the results. There is a need to also investigate the professionals’ perspective. Using the results at hand and conceptualizing the researchers’ as well as the professionals’ perspective will give a more comprehensive understanding of the area.

## Conclusion

This study brought together the experiences and knowledge of a wide range of researchers in one comprehensive and illustrative map of what involving professionals can lead to in research projects on ageing and health. The conceptual areas found in the study contribute to a knowledge basis for the understanding of professionals’ involvement in research on ageing and health. The conceptual areas also have significant implications for the theoretical development of involvement in research by illustrating the perspectives of process and outcome. In addition, the results of this study have implications for practice. The results could be of interest in developing instruments to measure involvement, and in developing education for both professionals and researchers. Also, the study is useful for improving the understanding of involvement in research and optimizing the involvement of professionals. However, the study also raises questions about the relation between process and outcome when involving professionals in research on ageing and health. Further research is required to explore this relationship. Moreover, further research into the professionals’ perspective is needed to obtain a more comprehensive understanding of the area and the differences and similarities of the two perspectives.

## Data Availability

The datasets used and/or analysed during the current study are available from the corresponding author on reasonable request.

## Abbreviations

BV: Bridging value
GCM: Group concept mapping
MDS: Multidimensional scaling
